# Communities of ground beetles (Carabidae, Coleoptera) in broad-leaved forests of protected and urban areas of the Kaluga Oblast (European Russia)

**DOI:** 10.3897/BDJ.8.e58688

**Published:** 2020-11-26

**Authors:** Maxim Shashkov, Sergei Alexeev, Natalya Ivanova

**Affiliations:** 1 Institute of Physicochemical and Biological Problems in Soil Science of Russian Academy of Sciences, Pushchino, Russia Institute of Physicochemical and Biological Problems in Soil Science of Russian Academy of Sciences Pushchino Russia; 2 Institute of Mathematical Problems of Biology RAS – the Branch of Keldysh Institute of Applied Mathematics of Russian Academy of Sciences, Pushchino, Russia Institute of Mathematical Problems of Biology RAS – the Branch of Keldysh Institute of Applied Mathematics of Russian Academy of Sciences Pushchino Russia; 3 State Budgetary Institution of the Kaluga Oblast "Parks Directorate", Biodiversity Conservation Department, Kaluga, Russia State Budgetary Institution of the Kaluga Oblast "Parks Directorate", Biodiversity Conservation Department Kaluga Russia

## Abstract

**Background:**

This sampling-event dataset provides primary data about species diversity, population and seasonal activity of ground beetles (Carabidae, Coleoptera). The study was carried out in broad-leaved forests of protected ("Kaluzhskiye Zaseki" Nature Reserve and Ugra National Park) and urban areas (the Kaluga City) of the Kaluga Oblast. Carabids were collected from April to October during 1995-1998 by pitfall traps. In total, 108,000 adult individuals of the Carabidae family were sampled; 105 species from 38 genera were counted.

**New information:**

This dataset is the first sampling-event dataset about the Carabidae family for the European part of Russia. It provides biodiversity data for new territory (Kaluga Oblast) and contributes to filling gaps in the global biodiversity distribution of the Carabidae family. Part of the data was collected from unique old-growth broad-leaved forests.

## Introduction

Carabidae is one of the most diverse insect families belonging to the largest order of Animalia, with over 40,000 described species (Lövei and Sunderland 1996, Bouchard et al. 2017). Family Carabidae has an almost cosmopolitan distribution, except arctic deserts and Antarctica. Being mostly broad polyphages, beetles of this family depend mainly on the entire set of biotic and abiotic factors specific to a particular geographic or natural region. Ground beetles often are indicators of specific ecosystems. The dependence on soil conditions is especially pronounced (Ghilarov 1965, Kryzhanovsky 1983). Considering their presence virtually in every terrestrial ecosystem, the ease of collection and identification of the most abundant species, ground beetles are a natural focus of entomological research. Carabidae beetles have been studied intensively by generations of coleopterists since the beginning of the XIXth century (Kryzhanovsky 1983), especially in Europe, including Russia (Gryuntal 2008, Kotze et al. 2011).

Unfortunately, most of the Russian data (especially collected in the Soviet period) was published in grey literature and not available for reuse. The most extensive carabid collection in Russia is stored in the Zoological Institute of Russian Academy of Sciences, Saint-Petersburg (Kryzhanovsky 1983), but not digitised, even at the level of metadata. Before our dataset publishing (30-08-2020), 2,534,360 occurrences of Carabidae beetles were published through Global Biodiversity Information Facility, GBIF.org (GBIF.org 2020a), but only 8139 amongst them derived from Russian territory (GBIF.org 2020b). The largest dataset (1934 records) for Russia includes occurrences of ground beetles in the north-east of European Russia (Konakova and Kolesnikova 2018). Other significant contributors to Russian Carabidae data are the iNaturalist citizen science project (Ueda 2012, 1357 records) and the Natural History Museum of the University of Tartu (the Natural History Museum University of Tartu and Abarenkov 2015, 1058 records). No sampling-event datasets about the Carabidae family were published through GBIF by Russian publishers. Contrary to long traditions of ground beetles investigations, Russia is still a gap on the global biodiversity map.

Data collected on the sampling plots, located on the urban district of Kaluga territory, were used in the writing of the monograph "Inventory of the Ground Beetles (Coleoptera, Carabidae) of Kaluga Urban Okrug" (Aleksanov and Alexeev 2019).

## Sampling methods

### Study extent

Kaluga Oblast is situated in the central part of the East European Plain. The distance between Kaluga City (administrative centre) and Moscow is about 150 kilometres, towards NNE. The climate is moderately continental with distinct seasons: warm and humid summers and cold winters with stable snow-cover (4-5 months). The mean annual precipitation of 600 mm and the mean annual temperature is about +4°C (RIHMI - WDC 2020). The total area covered by forests is around 1,380,000 ha (46% of the oblast territory). Agricultural lands occupied an area of 1,350,000 ha (44% of the territory).

**Figure 1.**
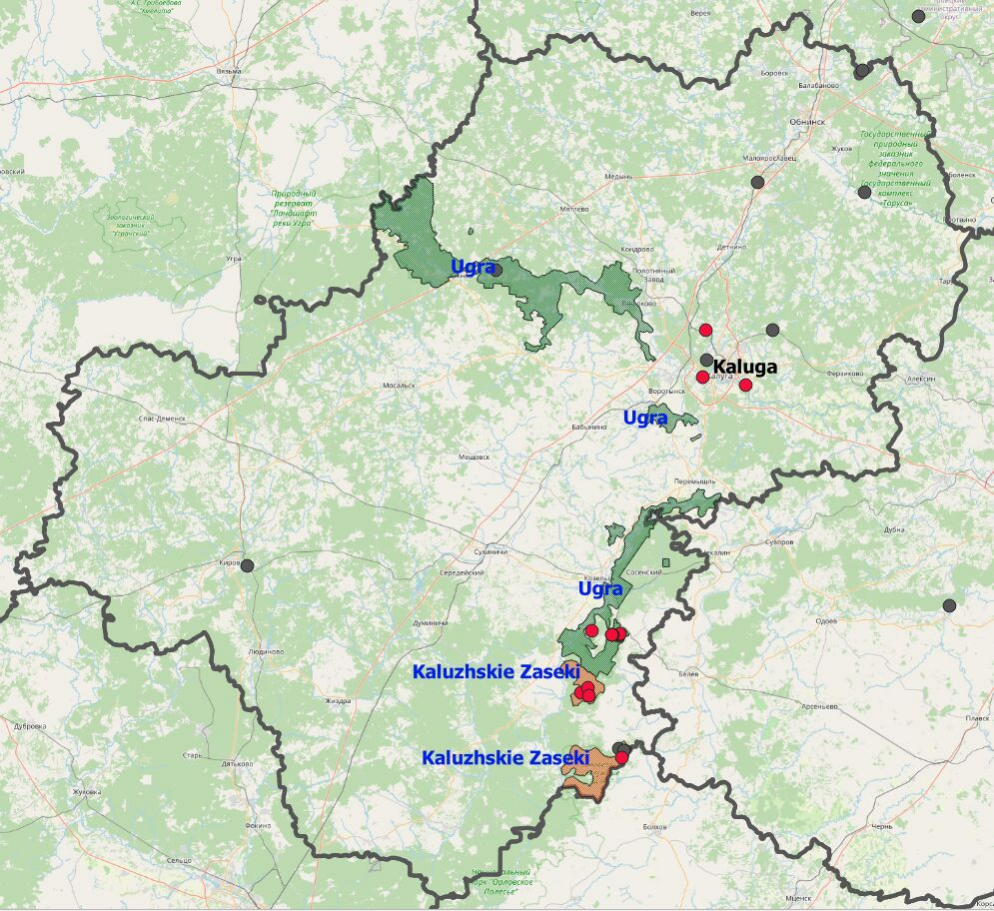
Study locations in the Kaluga Oblast: Ugra - Ugra National Park (green polygons), Kaluzhskiye Zaseki - "Kaluzhskiye Zaseki" Nature Reserve (orange polygons), Kaluga - Kaluga City. Grey dots - species occurrences according to GBIF.org 2020b, red dots - sampling plots.

Carabidae beetles were sampled from two nature protected areas and one urban territory (Fig. 1).

**Figure 2.**
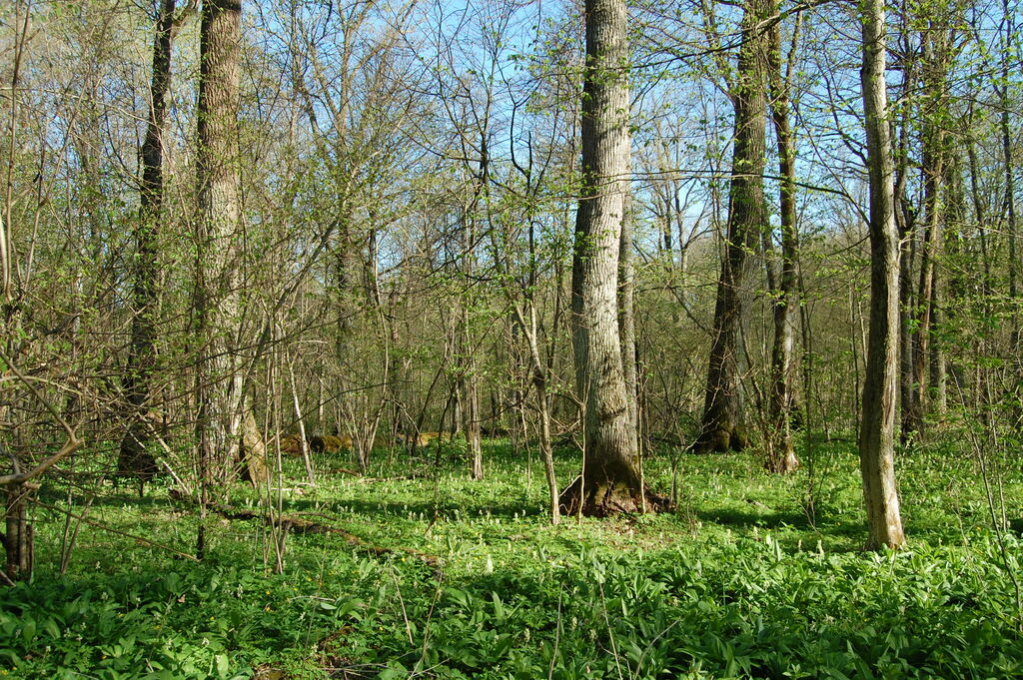
Broad-leaved forest in the "Kaluzhskiye Zaseki" Nature Reserve. Photo by Natalya Ivanova.

**Figure 3.**
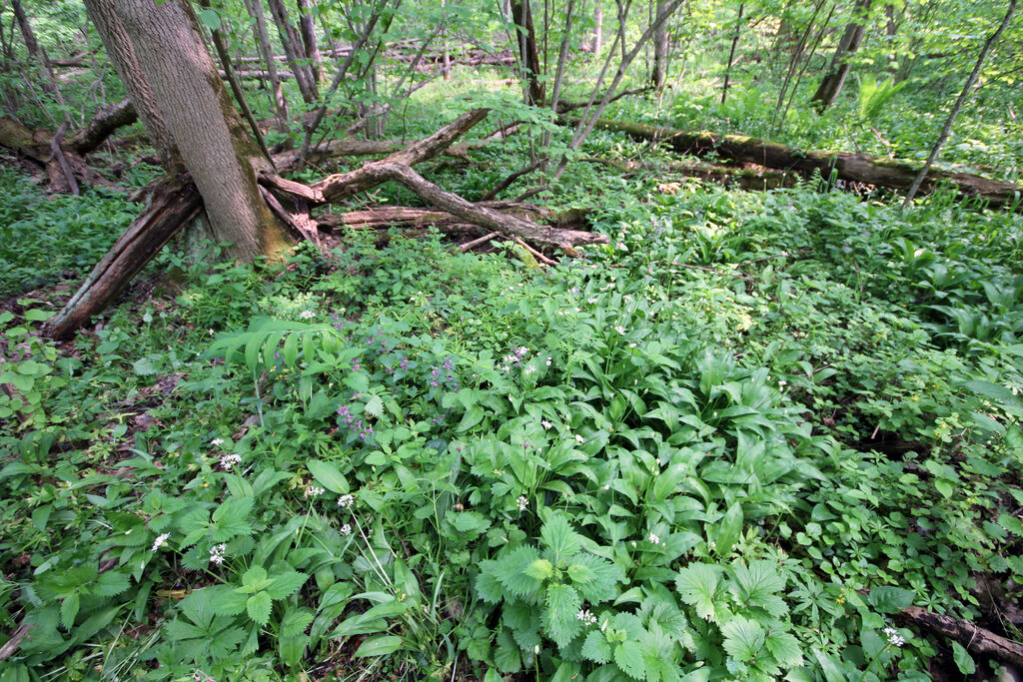
Broad-leaved forest in the "Kaluzhskie Zaseki" Nature Reserve. Photo by Maxim Shashkov.

"**Kaluzhskiye Zaseki" Nature Reserve**. The Reserve was established in 1992 due to the presence of unique old-growth broad-leaved forests that were mostly undisturbed by cutting and ploughing (Fig. 2 and Fig. 3) (Smirnova 1994). The total area of the Reserve is 185 km^2^. The Reserve consists of two separate parts, 12 km apart. Sampling plots were located in both clusters. The oldest patches of broad-leaved forests are remains of defence line against the raid of nomads and was under state protection for centuries.

**Figure 4.**
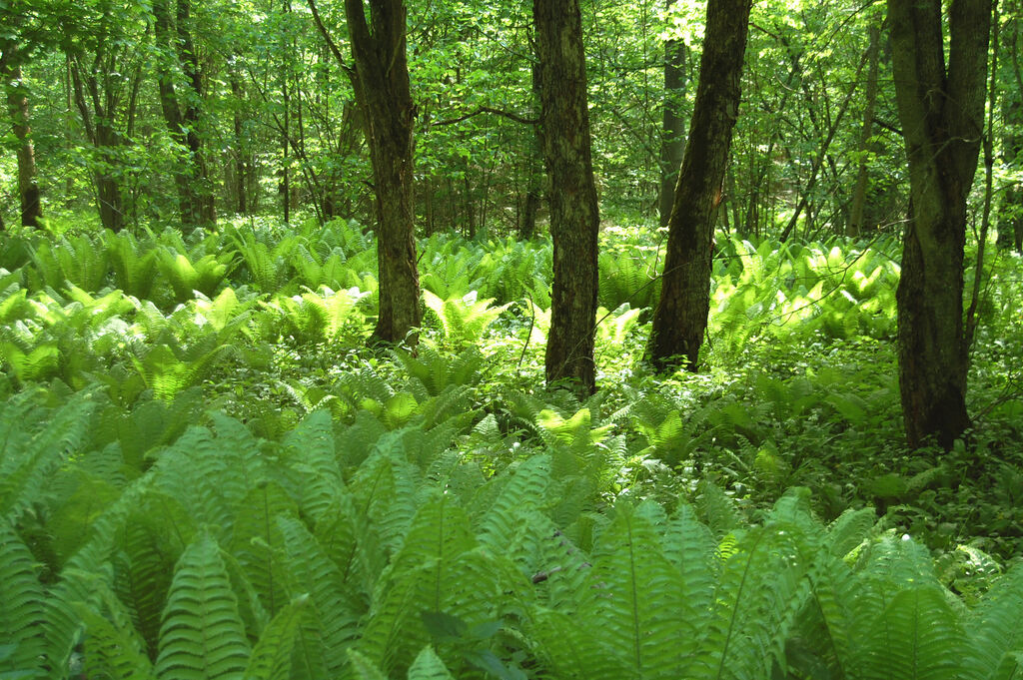
Broad-leaved forest in the Ugra National Park. Photo by Natalya Ivanova.

**Ugra National Park** was established in 1997 to protect typical landscapes of Central Russia and became a UNESCO biosphere reserve in 2002. The National Park consists of seven clusters grouped into three areas with a total area of 986,245 km^2^. This study was conducted in the Zhizdra cluster (Fig. 4).

**Kaluga City** is the administrative centre of Kaluga Oblast and a large industrial centre. The population is > 300,000 people. Carabidae beetles were sampled in three urban forest sites. These forests are not managed, with regrowth and understorey, but with noticeable signs of anthropogenic pressures.

All sampling plots were located in broad-leaved forests. According to FAO, soils of the sampling plots belong to the Luvisols group, except one belonging to Phaeozem. Textures of soils are sandy loam and silt loam, with soils of two sampling plots having a different texture - sandy loam on silt loam (moraine loam), the detailed description of which are represented in Table 1.

### Sampling description

Carabid beetles, alongside other epigeic arthropods, were sampled by soil pitfall traps (Greenslade 1964). On each forest site, 30 traps were installed, except one plot in 1995 with 90 traps. Traps were made of plastic bottles of 1.5 and 2 l volume cut at maximum diameter. The edge of the obtained cylinders was heated and folded inwards to ensure the rigidity of the trap. Sheds made of aluminium wire in the form of frames with transparent polyethene were used to protect traps. The type of traps and sheds on the one sampling site in 1995 was different: cylinders of plastic bottles, jars and plastic glasses for exploring the efficiency of carabids collecting (Alexeev and Aleksanov 2017) were used. Traps were filled with a solution of 1% formaldehyde for preserving the specimens and usually were sampled two times a month. Samples were sorted for carabids in the laboratory. Numerous and easily-recognisable species were identified by Maxim Shashkov and counted during sorting, others being stored on wadded pads for further identification.

Specimens of those species which were difficult to determine, were identified by Sergey Alexeev. Keys to Insects of the European Part of the USSR, vol. 2 (Gureva and Kryzhanovskii 1965) were used basically for identification of common, abundant species. The following keys were additionally used: Trautner and Geigenmueller 1987, Freude et al. 2004. The following were also used for some taxonomic groups: Isaev 2002, Kryzhanovsky 1983, Zherebtsov 2000, Yablokov-Khnzoryan 1976, Fedorenko 1992, Fedorenko 1993a, Fedorenko 1993b, Jacobson 1931. The identification of a number of specimens was checked by Igor Sokolov and Dmitry Fedorenko. The reference collection is kept in Kaluga at the personal disposal of Sergey Alexeev. Species names are given according to GBIF Backbone (GBIF Secretariat 2020).

## Geographic coverage

### Description

The European part of Russia, Kaluga Oblast. Locations of sampling plots are available in Table 2

### Coordinates

53.615 and 54.616 Latitude; 35.707 and 36.364 Longitude.

## Taxonomic coverage

### Taxa included

**Table:** 

Rank	Scientific Name	Common Name
family	Carabidae	Ground beetles (EN), Жужелицы (RU)

## Traits coverage

**Figure 5.**
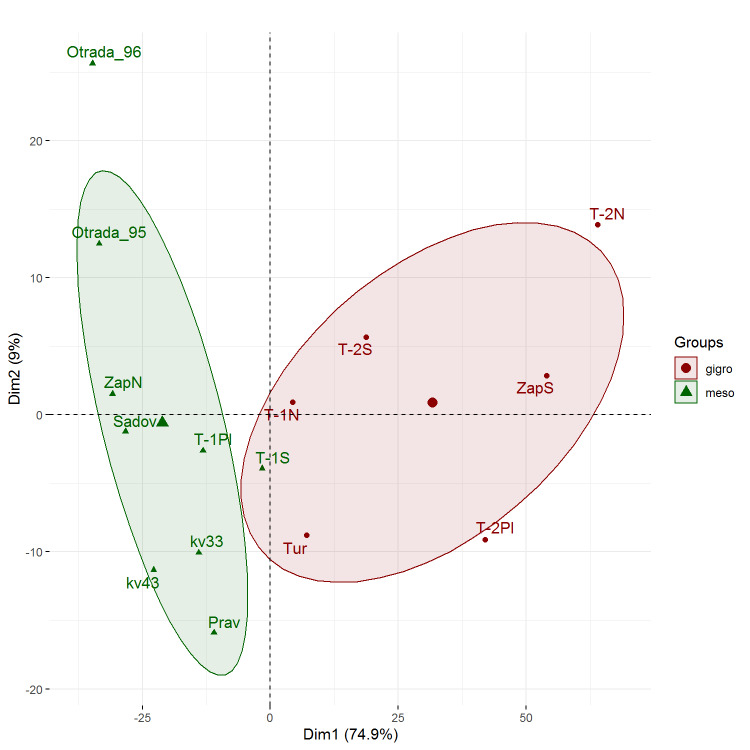
PCA ordination graph

During the exploratory data analysis, we performed principal component analysis (PCA) for 11 most abundant Carabid species (*Carabus granulatus* Linnaeus, 1758, *C. glabratus* Paykull, 1790, *C. hortensis* Linnaeus, 1758, *Cychrus caraboides* (Linnaeus, 1758), *Epaphius secalis* (Paykull, 1790), *Patrobus atrorufus* (Stroem, 1768), *Pterostichus niger* (Schaller, 1783), *P. aethiops* (Panzer, 1796), *P. oblongopunctatus* (Fabricius, 1787), *P. melanarius* (Illiger, 1798) and *Platynus assimile* (Paykull, 1790)). The first principal component explained 74.9% of the variation. For results interpretation, we checked a hydro-preferendum type as an important ecological factor, limited Carabid communities. Based on the relative species abundance, we assigned a hydro-preferendum type (xerophilic, mesophilic or hydrophilic) for the communities in each sampling plot. The preferendum for particular species was assigned according to the Eremin 1989, or the biotopic preferendum (Sharova 1982). We found sampling plots were divided into two groups on the graph according to the prevailing hydro-preferendum type (Fig. 5). The left (green) group combines mainly plots with coarse texture of soils (sandy loam and loamy sand), expected to be better drained and drier and the right contains plots on wetter soils with silt loam texture. These results revealed that humidity is a decisive factor for the studied carabid beetle communities.

## Temporal coverage

**Formation period:** 1995-5-25; 1996-4-20; 1997-4-20; 1998-4-30.

## Usage licence

### Usage licence

Other

### IP rights notes

Attribution 4.0 International (CC BY 4.0)

## Data resources

### Data package title

Communities of ground beetles (Carabidae, Coleoptera) in broad-leaved forests of protected and urban areas of the Kaluga Oblast (European Russia)

### Resource link

https://www.gbif.org/dataset/892a2c22-d234-4e74-a3b7-d1fb82fc731b

### Alternative identifiers

http://gbif.ru:8080/ipt/resource?r=carabidae_kaluga

### Number of data sets

1

### Data set 1.

#### Data set name

Communities of ground beetles (Carabidae, Coleoptera) in broad-leaved forests of protected and urban areas of the Kaluga Oblast (European Russia)

#### Data format

Darwin Core Archive format

#### Number of columns

29

#### Character set

UTF-8

#### Download URL

http://gbif.ru:8080/ipt/archive.do?r=carabidae_kaluga

#### Description

The dataset includes two related tables related by the eventID field – Events and Associated occurrences (Shashkov et al. 2020). The Event table consists of 18 fields, the Associated occurrences table - 11 fields. Occurrence table includes occurrence-present as well as occurrence-absent records.

**Data set 1.** 

Column label	Column description
eventID (Event Core)	An identifier for the trapping period https://dwc.tdwg.org/terms/#dwc:eventID
parentEventID (Event Core)	An identifier for the trap line https://dwc.tdwg.org/terms/#dwc:parentEventID
eventDate (Event Core)	Trap period (YYYY-MM-DD/YYYY-MM-DD) https://dwc.tdwg.org/terms/#dwc:eventDate
year (Event Core)	Year of data collection https://dwc.tdwg.org/terms/#dwc:year
startDayOfYear (Event Core)	The earliest integer day of the year on which the event occurred https://dwc.tdwg.org/terms/#dwc:startDayOfYear
endDayOfYear (Event Core)	The latest integer day of the year on which the event occurred https://dwc.tdwg.org/terms/#dwc:endDayOfYear
samplingProtocol (Event Core)	Sampling protocol https://dwc.tdwg.org/terms/#dwc:samplingProtocol
countryCode (Event Core)	The standard code for the Russian Federation according to ISO 3166-1-alpha-2 (RU) https://dwc.tdwg.org/terms/#dwc:countryCode
country (Event Core)	Country name https://dwc.tdwg.org/terms/#dwc:country
stateProvince (Event Core)	Region ("oblast") name. The first-level administrative division. https://dwc.tdwg.org/terms/#dwc:stateProvince
locality (Event Core)	The specific description of the place https://dwc.tdwg.org/terms/#dwc:locality
decimalLatitude (Event Core)	The geographic latitude in decimal degrees of the geographic centre of the data sampling place https://dwc.tdwg.org/terms/#dwc:decimalLatitude
decimalLongitude (Event Core)	The geographic longitude in decimal degrees of the geographic centre of the data sampling place https://dwc.tdwg.org/terms/#dwc:decimalLongitude
geodeticDatum (Event Core)	Spatial reference system (SRS) upon which the geographic coordinates are given in decimalLatitude and decimalLongitude as based https://dwc.tdwg.org/terms/#dwc:geodeticDatum
coordinateUncertaintyInMetres (Event Core)	The maximum uncertainty distance in metres https://dwc.tdwg.org/terms/#dwc:coordinateUncertaintyInMeters
coordinatePrecision (Event Core)	The fraction of a degree corresponding to the number of significant digits in the source coordinates https://dwc.tdwg.org/terms/#dwc:coordinatePrecision
habitat (Event Core)	Description of the habitat https://dwc.tdwg.org/terms/#dwc:habitat
samplingEffort (Event Core)	Amount of trap-days for each sampling term https://dwc.tdwg.org/terms/#dwc:samplingEffort
eventID (Occurrence Extension)	An identifier for the sampling term https://dwc.tdwg.org/terms/#dwc:eventID
occurrenceID (Occurrence Extension)	An identifier for the occurrence https://dwc.tdwg.org/terms/#dwc:occurrenceID
basisOfRecord (Occurrence Extension)	The specific nature of the record ("HumanObservation") https://dwc.tdwg.org/terms/#dwc:basisOfRecord
scientificName (Occurrence Extension)	Scientific name according to GBIF Backbone https://dwc.tdwg.org/terms/#dwc:scientificName
taxonRank (Occurrence Extension)	The taxonomic rank https://dwc.tdwg.org/terms/#dwc:taxonRank
identificationRemarks	Comments about previous identifications https://dwc.tdwg.org/terms/#dwc:identificationRemarks
occurrenceStatus (Occurrence Extension)	A statement about the presence or absence of this taxon in the trapping period https://dwc.tdwg.org/terms/#dwc:occurrenceStatus
organismQuantity (Occurrence Extension)	The quantity of beetles https://dwc.tdwg.org/terms/#dwc:organismQuantity
organismQuantityType (Occurrence Extension)	The type of quantification system used for the quantity of beetles (individuals per 100 trap days) https://dwc.tdwg.org/terms/#dwc:organismQuantityType
recordedBy (Occurrence Extension)	List of persons, who collected field data https://dwc.tdwg.org/terms/#dwc:recordedBy
identifiedBy (Occurrence Extension)	List of persons, who identified collected beetles https://dwc.tdwg.org/terms/#dwc:identifiedBy

## Figures and Tables

**Table 1.** Sampling plots description

Study area	Plot code	Habitat. Tree and herbs species dominants	Soil type / soil texture
“Kaluzhskiye Zaseki” Nature Reserve	ZapN	Broad-leaved forest. Tree stand: linden (*Tilia cordata* Mill.), oak (*Quercus robur* L.), ash (*Fraxinus excelsior* L.). Herb layer: *Carex pilosa* Scop.	Luvisol sod illuvial-ferruginous contact-gley / loamy sand
	ZapS	Old growth broad-leaved forest. Tree stand: *Tilia cordata*, *Fraxinus excelsior*, *Quercus robur*, aspen (*Populus tremula* L.). Herbs layer: *Matteuccia struthiopteris* (L.) Tod., *Allium ursinum* L. (in spring).	Phaeozem / silt loam
	kv33	Broad-leaved forest. Tree stand: Norway maple (*Acer platanoides* L.), *Tilia cordata*, *Quercus robur*. Herbs layer: *Mercurialis perennis* L., *Lamium galeobdolon* (L.) L., *Allium ursinum*.	Luvisol sod illuvial-ferruginous contact-gley / loamy sand
	kv43	Broad-leaved forest. Tree stand: *Acer platanoides*, *Quercus robur*, elm (*Ulmus glabra* Huds.), *Fraxinus excelsior*. Herbs layer: *Aegopodium podagraria*, *Galium odoratum* Scop., *Allium ursinum*.	Luvisol sod illuvial-ferruginous contact-gley on moraine loam / sandy loam on silt loam
Ugra National Park	T-1Pl	Broad-leaved forest. Tree stand: *Acer platanoides*, *Quercus robur*, *Fraxinus excelsior*, field maple (*Acer campestre* L.). Herbs layer: *Aegopodium podagraria*, *Mercurialis perennis*, *Allium ursinum*.	Luvisol grey forest / silt loam
	T-1N		
	T-1S		
	T-2Pl	Broad-leaved forest. Tree stand: *Fraxinus excelsior*, *Quercus robur*, *Acer platanoides*, *A. campestre*. Herbs layer: *Aegopodium podagraria*, *Mercurialis perennis*, *Allium ursinum*.	Luvisol grey forest slightly podzolics / silt loam
	T-2N	Broad-leaved forest. Tree stand: *Acer platanoides*, *Quercus robur*, *Tilia cordata*. Herbs layer: *Aegopodium podagraria*, *Lamium galeobdolon*, *Mercurialis perennis*, *Allium ursinum*.	
	T-2S	Broad-leaved forest. Tree stand: *Tilia cordata*, *Quercus robur*, *Acer platanoides*. Herbs layer: *Aegopodium podagraria*.	
	Otrada_95	Broad-leaved forest. Tree stand: *Tilia cordata*, *Quercus robur*, *Acer platanoides*. Herbs layer: *Carex pilosa*, *Aegopodium podagraria*.	Luvisol sod illuvial-ferruginous / loamy sand
	Otrada_96		
Kaluga city	Prav	Broad-leaved forest. Tree stand: *Tilia cordata*, *Quercus robur*, *Populus tremula*. Herb layer: *Lamium galeobdolon*, *Aegopodium podagraria*.	Luvisol grey forest / silt loam
	Sadov	Broad-leaved forest. Tree stand: *Populus tremula*, *Acer platanoides*, *Quercus robur*. Herbs layer: *Carex pilosa*.	Luvisol podbur illuvial-humic / sandy loam
	Tur	Broad-leaved forest. Tree stand: *Quercus robur*, *Tilia cordata*. Herbs layer: *Aegopodium podagraria*, *Convallaria majalis* L.	Luvisol podbur illuvial-ferruginous on moraine loam / sandy loam on silt loam

**Table 2.** Sampling plot locations

Study area	Plot code	Sampling year	Geographic coordinates (WGS 84)
“Kaluzhskiye Zaseki” Nature Reserve	ZapN	1996	53.76667, 35.70722
	ZapS	1996	53.61480, 35.86794
	kv33	1998	53.77861, 35.73500
	kv43	1998	53.76139, 35.73833
Ugra National Park	T-1Pl	1996	53.90222, 35.85972
	T-1N	1996	53.90444, 35.85917
	T-1S	1996	53.90583, 35.86167
	T-2Pl	1996	53.90417, 35.83333
	T-2N	1996	53.90333, 35.82861
	T-2S	1996	53.90361, 35.82889
	Otrada_95	1995	53.91333, 35.74833
	Otrada_96	1996	53.91333, 35.74833
Kaluga City	Prav	1997	54.50556, 36.19333
	Sadov	1997	54.61559, 36.20463
	Tur	1997	54.48750, 36.36361
